# Genetic Alterations and Resectability Predict Outcome in Patients with Neuroblastoma Assigned to High-Risk Solely by *MYCN* Amplification

**DOI:** 10.3390/cancers13174360

**Published:** 2021-08-28

**Authors:** Frank Berthold, Angela Ernst, Sandra Ackermann, Christoph Bartenhagen, Holger Christiansen, Barbara Hero, Carolina Rosswog, Dietrich von Schweinitz, Thomas Klingebiel, Irene Schmid, Thorsten Simon, Matthias Fischer

**Affiliations:** 1 Department of Pediatric Oncology and Hematology, University of Cologne, Kerpener Str. 62, 50924 Cologne, Germany; barbara.hero@uk-koeln.de (B.H.); thorsten.simon@uk-koeln.de (T.S.); 2 Institute of Medical Statistics and Computational Biology, Medical Faculty, University of Cologne, 50924 Cologne, Germany; angela.ernst@uk-koeln.de; 3 Center for Molecular Medicine Cologne (CMMC) and Department of Experimental Pediatric Oncology, Medical Faculty, University of Cologne, Kerpener Str. 62, 50924 Cologne, Germany; sandra.ackermann@uk-koeln.de (S.A.); c.bartenhagen@uni-koeln.de (C.B.); carolina.rosswog@uk-koeln.de (C.R.); matthias.fischer@uk-koeln.de (M.F.); 4 Department of Pediatric Oncology, Hematology and Hemostasiology, University of Leipzig, Liebigstr. 20a, 04103 Leipzig, Germany; holger.christiansen@medizin.uni-leipzig.de; 5 Else Kröner Forschungskolleg Clonal Evolution in Cancer, University Hospital Cologne, Kerpener Str. 62, 50924 Cologne, Germany; 6 Department of Pediatric Surgery, von Hauner Children’s Hospital, Ludwig-Maximilians-University Munich, Lindwurmstr. 4, 80337 München, Germany; dietrich.schweinitz@med.uni-muenchen.de (D.v.S.); irene.schmid@med.uni-muenchen.de (I.S.); 7 Department of Children and Adolescents, University Hospital, Goethe University Frankfurt, Theodor-Stern-Kai 7, 60596 Frankfurt, Germany; thomas.klingebiel@kgu.de

**Keywords:** high-risk neuroblastoma, *MYCN* amplification, *RAS* pathway, p53 pathway, *ALK*, resectability

## Abstract

**Simple Summary:**

Currently, patients with high-risk neuroblastoma are uniformly treated with maximum therapy. This study investigated a high-risk subgroup characterized by the presence of the amplified *MYCN* oncogene in the tumor regardless of the stage. In contrast to the corresponding high-risk subgroup consisting of patients with metastases and age at diagnosis over 18 months, the investigated subgroup had generally a superior survival chance. However, the detection of mutations of specific genes in the tumor tissue (RAS and p53 pathway including ALK) had a strong, negative impact. These genes should be therefore also investigated in the future. Complete surgical removal of the primary tumor proved to be beneficial for high-risk neuroblastoma patients assigned to the high-risk category solely by *MYCN* amplification.

**Abstract:**

Background: To identify variables predicting outcome in neuroblastoma patients assigned to the high-risk group solely by the presence of *MYCN* oncogene amplification (MNA). Methods: Clinical characteristics, genomic information, and outcome of 190 patients solely assigned to high-risk neuroblastoma by MNA were analyzed and compared to 205 patients with stage 4 neuroblastoma aged ≥18 months with MNA (control group). Results: Event-free survival (EFS) and overall survival (OS) at 10 years were 47% (95%-CI 39–54%) and 56% (95%-CI 49–63%), respectively, which was significantly better than EFS and OS of the control group (EFS 25%, 95%-CI 18–31%, *p* < 0.001; OS 32% 95%-CI 25–39%, *p* < 0.001). The presence of *RAS-*/p53-pathway gene alterations was associated with impaired 10-year EFS and OS (19% vs. 55%, and 19% vs. 67%, respectively; both *p* < 0.001). In time-dependent multivariable analyses, alterations of RAS-/p53-pathway genes and the extent of the best primary tumor resection were the only independent prognostic variables for OS (*p* < 0.001 and *p* = 0.011, respectively). Conclusions: Neuroblastoma patients attributed to high risk solely by *MYCN* amplification have generally a more favorable outcome. Mutations of genes of the RAS and/or p53 pathways and incomplete resection are the main risk factors predicting poor outcome.

## 1. Introduction

Neuroblastoma is considered as high-risk if the calculated 5-year event-free survival is below 50% for de novo patients. To this category belong patients with distant metastases aged ≥18 months at diagnosis (stage 4/M) and those with *MYCN* amplification (MNA) regardless of their stage (1–3, 4S/MS, 4/M) and age [1,2,3]. The significance of MNA for unfavorable outcomes has been demonstrated in patients with localized neuroblastoma (stages 1, 2, 3/L1–2) [4,5,6,7,8], stage 4S/MS patients [9,10], and also stage 4/M patients aged less than 18 months at diagnosis [2,11,12]. Thus, there is strong evidence that patients with *MYCN*-amplified neuroblastoma who are otherwise at low or intermediate risk have an inferior prognosis compared to those without MNA.

In the literature, the mentioned criteria are not strictly observed. Studies on outcome of high-risk neuroblastoma patients commonly report two categories, i.e., those diagnosed as stage 4 over the age of 18 months (major group) and those assigned to high risk due to other criteria (minor group). Various definitions of the minor group using distinct criteria, e.g., MNA, stage 3 without MNA, unfavorable histology, and unfavorable ploidy, have resulted in variable sizes and limited comparability of minor groups in different studies. Nonetheless, the outcome of these patients was generally more favorable compared to the major group, although outcomes were rarely reported separately [4,5,6,8,11,12,13], impeding comparisons of results from different trials for high-risk neuroblastoma.

The impact of the varying definitions and conditions is highlighted by the role of surgery in high-risk neuroblastoma. The SIOPEN group reported an improved survival for stage 4 patients who responded to induction chemotherapy and had complete macroscopical excision of the primary tumor (plus high-dose chemotherapy, local radiotherapy and immunotherapy) [14]. In another smaller study, stage 4 patients with poor response to induction chemotherapy had no outcome benefit from a higher extent of primary tumor resection [15]. The German group found no impact of the extent of surgical resection on outcome in high-risk stage 4 patients aged over 18 months [16]. A systematic review and meta-analysis demonstrated some advantage for gross total resection regarding disease-free survival; however, OS was not improved in stage 4 disease [17]. The time point of the surgical intervention (at diagnosis vs. delayed after induction chemotherapy) may also play a role (reflecting the grade of initial resectability?) [18]. These partially conflicting results from different studies and different cohorts suggest that subgroup investigations on the role of surgery may be meaningful.

It has recently been demonstrated that the presence of telomere maintenance mechanisms and mutations of genes of the RAS- and p53-pathway are major determinants of clinical courses in neuroblastoma [19,20]. Telomere maintenance may result from various genomic alterations, including MNA [21]. It has remained unclear, however, whether mutations in the pre-defined set of genes related to the RAS-/p53-pathway [19] impact clinical outcomes of the subgroup of children with MNA but otherwise low- or intermediate-risk characteristics.

Thus, we here aimed to examine the clinical and genetic characteristics together with the effect of time-dependent and -independent therapeutic interventions on the outcome of neuroblastoma patients considered high risk solely due to MNA.

## 2. Patients and Methods

Inclusion criteria for the study group were (i) neuroblastoma with INSS stages 1, 2, or 3 [22] of all ages, or stage 4S, or stage 4 aged <18 months at diagnosis; (ii) *MYCN* amplification (>4-fold *MYCN* copy number in relation to the copy number of chromosome 2 [23]); (iii) registration in the trial NB97 (high-dose arm, as treated) or the NB2004-HR trial (experimental and standard arm [24]); and (iv) diagnosis between 5 March 1997 and 31 December 2016. Exclusion criteria were (i) second malignancy after neuroblastoma diagnosis, (ii) withdrawal of consent for data use, and (iii) loss to follow-up before first chemotherapeutic course.

High-risk patients with stage 4 aged ≥18 months at diagnosis with MNA served as the control group. All other inclusion and exclusion criteria were identical to the study group.

Patients were treated according to the high-risk strata of trials NB97 and NB-2004-HR [13]. The high-dose arm of trial NB97 was adopted as the standard in the NB2004-HR protocol [25], and outcome results of the two NB2004-HR arms were identical. Therefore, treatment efficacy was considered to be identical between the high-dose arm of NB97 and the two arms of NB2004-HR. Anti-GD2 antibody therapy was included during the period 1997–2002 of the NB97 trial. Afterwards, antibody therapy was not protocol treatment until 2016 [24].

The primary endpoint was event-free survival (EFS). The analyses are regarded as exploratory, and *p*-values are given as descriptive measures to detect meaningful effects. EFS was defined as the time from the date of diagnosis until recurrence or progress or until death of any cause or until the last examination. Overall survival (OS) was calculated from the date of diagnosis until death of any cause or until the last examination. Kaplan–Meier estimates for EFS and OS were compared by log-rank tests or the Fleming–Harrington *p*-value if the proportional hazards assumption did not hold.

Univariable analysis was used to investigate the individual prognostic impact of risk factors, which are indicated in Table 1. For multivariable survival analyses the Cox regression model was used. After testing for multicollinearity, included variables were chromosome 1p aberrations, primary tumor site (cervical, thoracic, adrenal, abdominal non-adrenal, unknown, >1 site, combined regions, >1 site multilocular), initial number of metastatic organs), sex, age, stage, best result of surgical resection regardless of the time point and the number of operations (at diagnosis or after preceding chemotherapy, first or second look surgery), lactic dehydrogenase (LDH), ferritin, homovanillic acid (HVA) and/or vanillylmandelic acid (VMA), neuron-specific enolase (NSE), percutaneous radiotherapy, antibody therapy, metaiodinebenzylguanidine (mIBG) therapy, high-dose chemotherapy with autologous stem cell support (ASCT), and RAS and/or p53 pathway alterations. The covariates were fitted into a stepwise model selection process (forward and backward with Akaike Information Criterion (AIC) to select the better fitting model) and the proportional hazards assumption tested in the final models. The likelihood ratio test *p*-value for inclusion was ≤0.05 and for exclusion >0.10. Estimated hazard ratios (HR) with 95% confidence intervals (95% CI) and Wald *p*-values were calculated. For all analyses, IBM SPSS statistical package version 26 and R version 4.0.3 were used. The data lock for this analysis was 24 March 2020.

Chromosome 1p aberrations and copy numbers of the oncogene *MYCN* were investigated twice per tissue sample by two independent laboratories and analyzed according to the international consensus [23,26]. All tumors of the study cohort were considered to be telomerase-positive because telomerase-reverse-transcriptase (*TERT)* is transcriptionally activated by *MYCN,* as demonstrated previously [21,27]. Genes related to the RAS and p53 pathways were defined according to Ackermann et al. [19] and included ALK as an upstream activator of the RAS-MAPK pathway. Genomic alterations affecting these genes were determined by massively parallel sequencing, as described previously [19]. Of the 87 study patients, 56 were already reported (with less corresponding clinical details) [19].

## 3. Results

**Figure 1:**
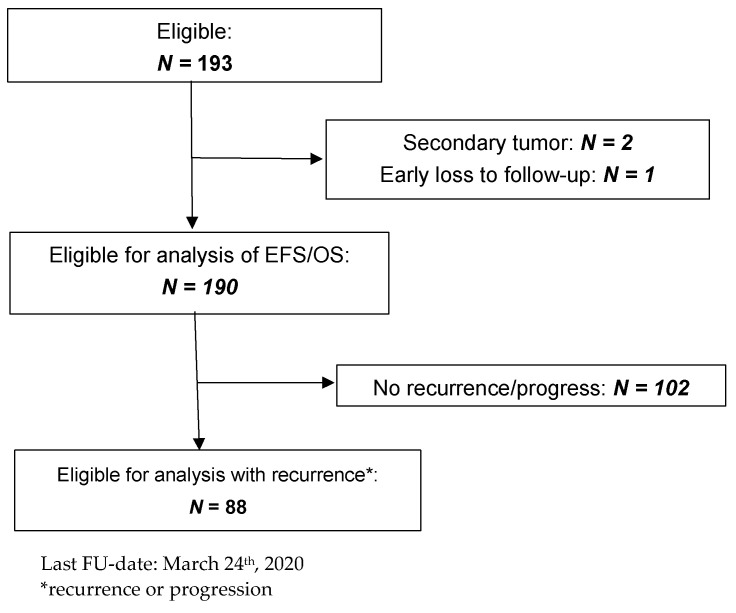
CONSORT diagram.

Within the investigation period, 823 patients with high-risk neuroblastoma were enrolled in the trials NB97 (high-dose chemotherapy arm) and NB2004 (both high-risk arms). Of these, 193 (23.5%) were considered as high risk solely by MNA. Three patients were excluded (Figure 1), resulting in 190 patients for analysis, referred to as the study group. The control group consisted of 205 high-risk patients aged ≥18 months with stage 4 disease and MNA.

### 3.1. Characteristics of the Patients and Tumors

Of the 190 study patients, 93 had localized disease (49%), 26 had stage 4S (14%), and 71 (37%) had stage 4 and were <18 months at diagnosis (Table 1). Study patients had less frequently elevated tumor markers and unfavorable histology [28] compared to the control group (Table 1). Patients with stage 4 aged <18 months had more frequent liver metastasis compared to those aged ≥18 months at diagnosis (28% vs. 12%).

The frequency of losses at the short arm of chromosome 1 did not differ between tumors of the study and control group. Information on genomic alterations of genes of the RAS and p53 pathway [19] was available from 87 patients of the study group and from 49 patients of control group. In the study group, the outcome of patients with and without such information did not differ (*p* = 0.612 for EFS and *p* = 0.633 for OS), indicating that there was no systematic selection bias between these cohorts. The same was true for the control group (*p* = 0.590 for EFS and *p* = 0.083 for OS). Mutations in RAS/p53 pathway-related genes tended to occur less frequently in the study than in control group (24% (21/87 cases) vs. 39% (19/49 cases)), with the majority of alterations affecting *ALK* both in the study and control group (Table 2).

### 3.2. Treatment and Overall Outcome

**Figure 2:**
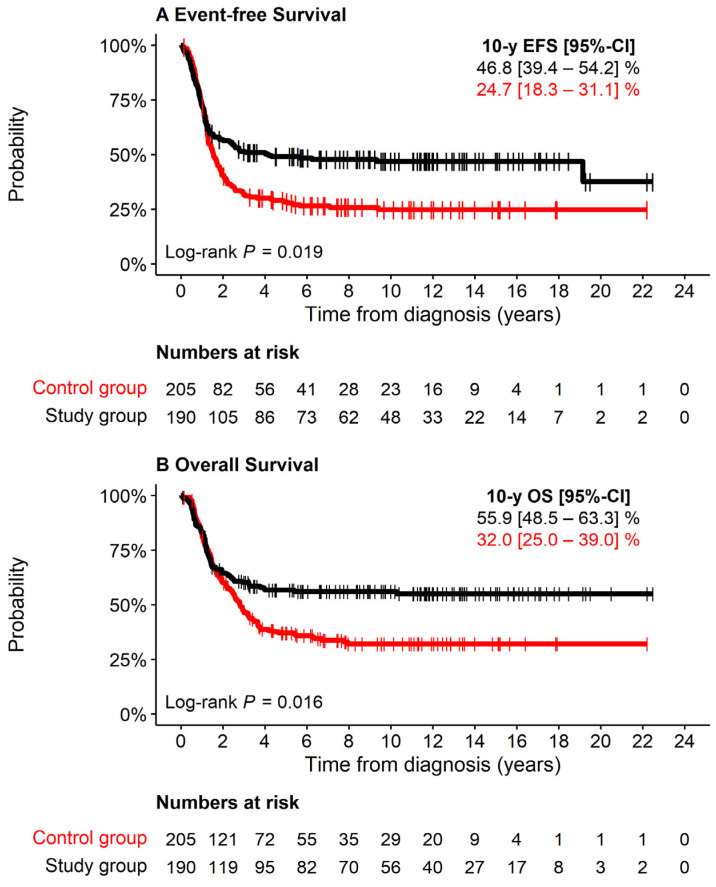
EFS and OS of 190 study patients attributed to high-risk solely due to MNA (black), 205 patients with stage 4 aged >18 months at diagnosis and MNA (control group, red). (**A**) Event-free Survival. (**B**) Overall Survival.

Eighty percent of the study group had received high-dose chemotherapy with autologous stem cell support. Infants <6 months at diagnosis had been excluded by protocol. Sixteen percent received antibody treatment, 10% ^131^mIBG therapy, and 7% percutaneous irradiation. The 10-year EFS of the study group was 47% (95% CI 39–54%), and 10-year OS was 56% (95% CI 49–63%), which was significantly better than that of the control group (10-year EFS 25%, 95% CI 18–31%; 10-year OS 32%, 95% CI 25–39%, both log-rank *p* < 0.001) (Figure 2). In both groups death was caused by tumor progression in 87%, while 13% of deaths were due to treatment toxicity or could not be distinguished between tumor progression and toxicity (Table 1).

### 3.3. Impact of Clinical and Biological Characteristics on Outcome in the Study Group

**Figure 3:**
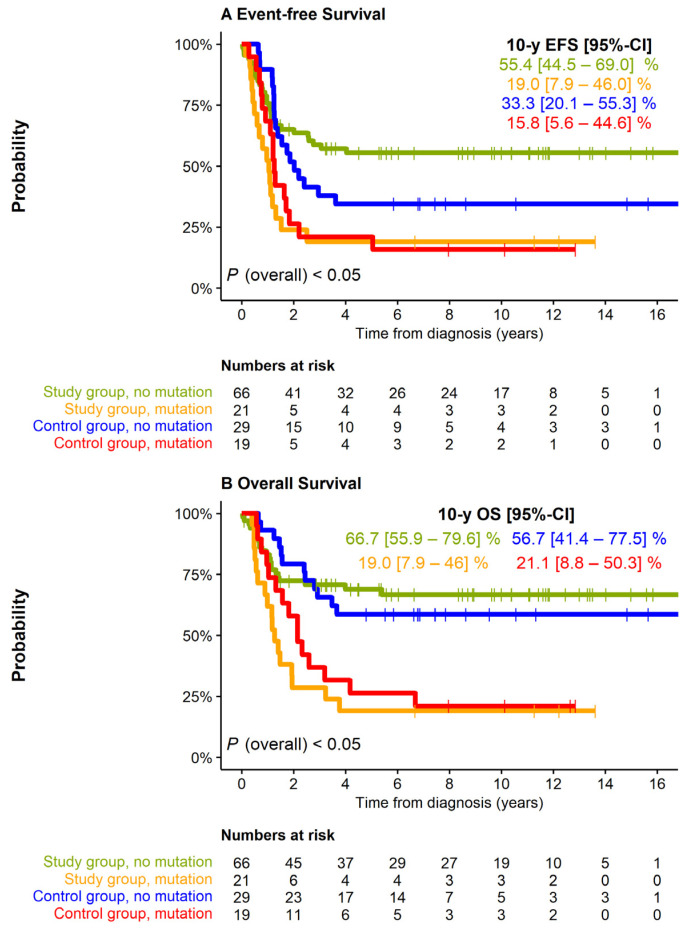
EFS and OS of 87 study group (*N* = 87) and control group (*N* = 39) patients by the presence of mutations in the genes of the *RAS* and p53 pathways (including ALK). (**A**): EFS, (**B**): OS. Pairwise comparisons (Log-rank *p*-values, Bonferroni–Holm adjusted). EFS: Study group, no mutation vs. study group, mutation: 0.003. Study group, no mutation vs. control group, no mutation: N.A. (lines cross). Study group, no mutation vs. control group, mutation: 0.012. Study group, mutation vs. control group, no mutation: 0.028. Study group, mutation vs. control group, mutation: 0.510. Control group, no mutation vs. control group, mutation: 0.094. OS: Study group, no mutation vs. study group, mutation: <0.001. Study group, no mutation vs. control group, no mutation: N.A. (lines cross). Study group, no mutation vs. control group, mutation: 0.003. Study group, mutation vs. control group, no mutation: 0.001. Study group, mutation vs. control group, mutation: 0.393. Control group, no mutation vs. control group, mutation: 0.013.

Study group patients with stage 4 disease had an inferior 10-year EFS and tended to have a lower OS in comparison to patients with stages 1–3 or 4S (log-rank *p* = 0.038 and *p* = 0.077, respectively; Supplementary Figure S1). No impact on EFS of the study group was observed for the characteristics age at diagnosis (independent of the age threshold 12, 18, or 24 months, *p* = 0.672, *p* = 0.058, *p* = 0.230, respectively), elevation of catecholamine metabolites (VMA/HVA) or ferritin (log-rank *p* = 0.541 and *p* = 0.865, respectively), and chromosome 1p status (normal vs. imbalance, log-rank *p* = 0.635; normal vs. deletion, *p* = 0.660; imbalance vs. deletion, *p* = 0.659). By contrast, mutations of RAS or p53 pathway genes were strongly associated with an unfavorable outcome (10-year EFS, 19% vs. 55%, 10-year OS, 19% vs. 67%, both *p* < 0.001; Figure 3). Patients whose tumors harbored *ALK* mutations had similarly poor outcome as those whose tumors harbored alterations of other RAS or p53 pathway-related genes (*p* = 0.624 for EFS and *p* = 0.425 for OS). In the control group a similar, albeit less pronounced, prognostic effect of mutations in RAS-/p53 pathway-related genes was observed (10-year EFS 16% vs. 33%, log-rank *p* = 0.063; 10-year OS 21% vs. 57%, log-rank *p* = 0.008).

### 3.4. Therapy-Related Risk Factors

**Figure 4:**
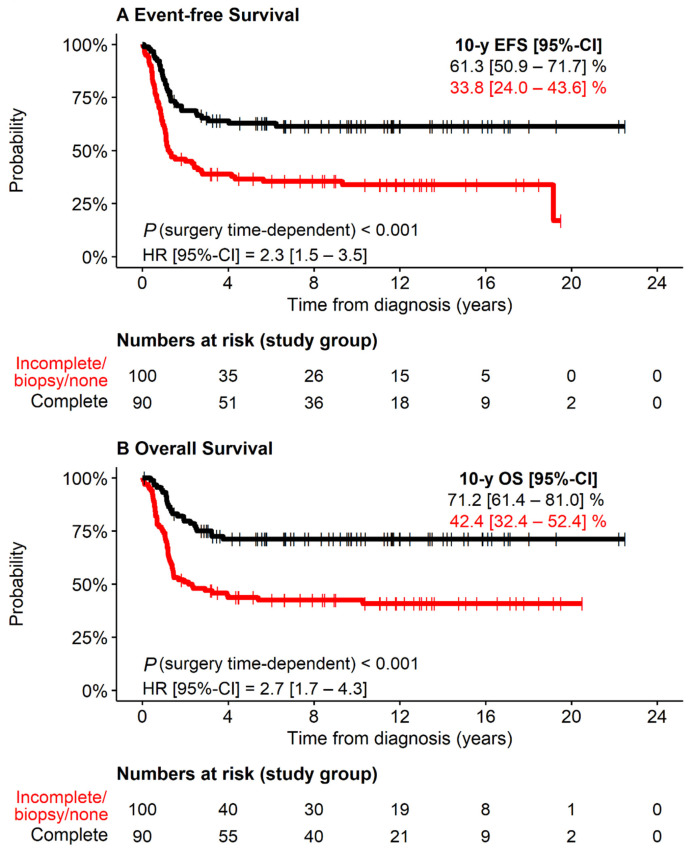
EFS and OS of 190 study group patients with *MYCN* amplification and stages 1, 2, 3, 4S or stage 4 aged <18 months by completeness of surgical resection *. * If more than one surgical intervention, the best result was counted. (**A**) Event-free Survival. (**B**) Overall survival.

Patients with macroscopically complete resection of the primary tumor (47% of patients) had both better EFS and OS than those with incomplete resection (35%), biopsy only (10%), or no surgical intervention (8%; Figure 4). Within the group of patients with incompletely resected primary tumors, the outcome was not different between biopsy and macroscopically incomplete resection (log-rank EFS *p* = 0.989, log-rank OS *p* = 0.629). Since stage 1 corresponds to macroscopically complete resection by definition, we excluded these patients (*N* = 5). In the remaining stage-defined groups, macroscopically complete resection was associated with better outcome in stage 3 patients (*p* < 0.001 for EFS, *p* < 0.001 for OS; *N* = 68), stage 4S patients (*p* = 0.013 for EFS, *p* = 0.006 for OS; *N* = 26), and stage 4 patients aged <18 months (*p* = 0.056 for EFS, *p* = 0.012 for OS; *N* = 71), while it was not prognostic in stage 2 patients (*p* = 0.685 for EFS, *p* = 0.553 for OS; *N* = 20; Supplementary Figure S2).

High-dose chemotherapy was associated with both better EFS and OS (likelihood ratios *p* < 0.001), while antibody therapy was not (likelihood ratio EFS *p* = 0.133; likelihood ratio OS *p* = 0.083). Intravenous systemic ^131^mIBG radiotherapy and percutaneous irradiation were given only in the case of active residual tumor following induction chemotherapy, and they were associated with similar (likelihood ratio *p* = 0.459) and worse outcome (likelihood ratio *p* = 0.026), respectively, compared to non-irradiated patients.

### 3.5. Definition of Risk Groups

To define risk groups within the study group, we performed multivariable analysis based on EFS and OS. The multivariable model based on EFS revealed that ‘mutation in RAS-/p53 pathway genes’ was the only independent prognostic marker for poor outcome, whereas both ‘mutation in RAS-/p53 pathway genes’ and ‘less than complete resection of the primary tumor’ were independent prognostic markers for poor OS (Table 3; Supplementary Figure S3). Notably, OS of patients with complete resection of the primary tumor and no mutation, who comprised more than one-third of the cohort, was >80%. When the mutation status was not available, the variables ‘stage’ and ‘best surgery’ were independent prognostic markers for EFS as well as OS (Table 3).

### 3.6. Tumor Recurrences

Tumor recurrences were observed in 88 patients after a median time of 12 months (Table 4), which was shorter than in the control group (15 months, *p* = 0.003). The main recurrence sites were the primary site (66%), osteomedullary (35%), central nervous system (20%), liver (20%), and lymph nodes (15%). Forty-five percent of the relapsed patients had recurrences in more than one site. Patients in the control group more frequently had osteomedullary recurrences (58%, *p* = 0.001) and at the primary site (41%, *p* = 0.036).

Eleven of the ninety-three patients with localized neuroblastoma and 11/26 stage 4S patients progressed to stage 4 (12% and 42%, respectively), mostly representing a spread to multiple organs (19/22 cases). Patients with localized progression had a better secondary EFS (log-rank *p* = 0.013) and OS (log-rank *p* = 0.010) compared to those with metastatic progression. The median times from the first to next recurrence or death were short (interval first to second recurrence, 2.2 months, 95% CI, 1.8–2.5; interval first recurrence to death, 4.5 months, 95% CI, 3.6–5.4).

## 4. Discussion

We here demonstrate that the outcome of neuroblastoma patients who are considered high-risk only due to MNA is substantially better than that of other high-risk patients. We also found that alterations of genes of the RAS and the p53 pathways were strong prognostic markers in this patient cohort: If such mutations were present in the tumor, the outcome of patients was very poor, whereas the majority of patients survived if such mutations were absent. Since several of the detected alterations represent actionable targets, particularly mutated *ALK* [29,30], we suggest that integration of targeted therapies into frontline treatment should be considered in this patient subgroup in the future. In addition, complete resection of the primary tumor was an independent prognostic marker for the entire study cohort and, most importantly, also within subgroups defined by the stages 3, 4S, and 4. While it remains speculative whether this prognostic effect is due to surgery itself, response to chemotherapy, and/or differences in the biology of completely and incompletely resected tumors, it is a clinically relevant finding that may guide treatment decisions in these patients in future clinical trials. Nonetheless, the data indicate that complete resection of the primary tumor is likely to improve outcome in this specific cohort of patients and surgeons encouraged accordingly (without increasing the risk for life and organs). If information on genetic alterations was not available, stage was an independent prognostic marker, in addition to complete resection. Together, our data suggest that alterations of a defined set of RAS and p53 pathway-related genes should be determined at the time of diagnosis in these patients to assess their individual risk, and that complete resection should be aimed for in the treatment concept.

The 10-year EFS and OS of 47% and 56%, respectively, in the study group is well in line with other reports on localized neuroblastoma with *MYCN* amplification. Bagatell et al. reported a 5-year EFS and OS of 53 ± 8% and 72 ± 7%, respectively, for stage 1 and 2 patients with MNA tumors, compared to 90 ± 1% and 98 ± 1%, respectively, in patients with non-amplified tumors (*n* = 8800 patients, collected in the international INRG database) [4]. *MYCN* amplification was also predictive for a poorer outcome in stage 3 patients of the same cohort (5-year EFS and OS with *MYCN* amplification 45 ± 4% and 48 ± 4%, respectively, vs. 81 ± 1% and 89 ± 2% without *MYCN* amplification, respectively) [5]. Since Germany contributed to the international cohort collected in the INRG database, a certain overlap between the reported groups should be considered. In line with these figures, the SIOPEN group reported 71% 5-year EFS for stage 2 and 3 MNA neuroblastoma [31]. Similar to these observations, two additional studies reported a poorer outcome in small cohorts of stage 3 patients when *MYCN* amplification was present in the tumors [6,7]. EFS and OS at 5 years was also lower in 6 infants with localized MNA neuroblastoma (50% and 67%, respectively) in comparison to 340 children without MNA (95% and 98%, respectively) in a Japanese study [8].

The strengths of this study are (i) the fact that 99% of patients diagnosed during the study periods were covered [13], (ii) the availability of corresponding clinical and molecular characteristics, and (iii) a largely uniform and modern treatment schedule. It has to be noted, however, that the treatment regimens applied to patients were not completely identical, although these differences had no impact on patient outcome in the respective trials [24,25]. Another potential limitation of the study is the fact that the results have been obtained retrospectively and should thus be considered as descriptive (although data had been collected prospectively). We therefore suggest to validate our findings in a prospective study. Finally, the Cox model estimates may be unstable due to the low number in some groups

## 5. Conclusions

Our study demonstrates that RAS and p53 pathway alterations have a strong impact on outcome of neuroblastoma patients assigned to high-risk solely by the presence of MNA. We therefore suggest to complement *MYCN* diagnostics by investigating genetic alterations of a predefined set of genes related to the RAS and p53 pathways. For routine clinical care, stage and resectability of the primary tumor are the important risk factors, and information on these is always available.

## Figures and Tables

**Table 1:** Patient characteristics.

Characteristic	Study Group	Control Group	*p*
	Stages 1, 2, 3, 4S, 4 < 18 m	Stage 4 ≥ 18 m and MNA	
	*N* (%)	*N* (%)	
**All**	190 (100)	205 (100)	
**Sex**	190 (100)	205 (100)	
Male	111 (58)	124 (60.5)	0.683
Female	79 (42)	81 (39.5)	
**Age at diagnosis (m)**	190 (100)	205 (100)	
Median (inter-quartile range)	14.3 (9.0–26.0)	30.5 (24.1–43.9)	<0.001 **
**Primary tumor site**	190 (100)	205 (100)	
Abdominal adrenal	132 (70)	143 (70)	>0.999
Abdominal non-adrenal	53 (28)	59 (29)	0.911
Thoracic	12 (6)	18 (9)	0.448
Cervical	1 (1)	2 (1)	N.A.
Unknown	0 (0)	0 (0)	N.A.
>1 site (combined regions)	8 (4)	14 (7)	0.280
>1 site (multilocular)	8 (4)	3 (2)	0.128
**Stage (INSS)**	190 (100)	205 (100)	
1, 2, 3 or 4S	119 (63)	0 (0)	N.A.
1	5 (3)	0 (0)	N.A.
2	20 (10)	0 (0)	N.A.
3	68 (36)	0 (0)	N.A.
4S	26 (14)	0 (0)	N.A.
4 < 18 m	71 (37)	0 (0)	N.A.
4 ≥18 m	0 (0)	205 (100)	N.A.
**Sites of initial metastasis stage 4**	Stage 4 < 18 m 71 (100)	Stage 4 ≥ 18 m and MNA 205 (100)	
Bone marrow (cytology)	65 (92)	189 (92)	0.804
Osteomedullary (mIBG scintigraphy)	67 (94)	196 (96)	0.746
Lymph nodes	20 (28)	55 (27)	0.877
Liver	20 (28)	24 (12)	**0.002**
Brain/spinal cord	2 (3)	9 (4)	0.734
Lung/pleura	8 (11)	16 (8)	0.463
Skin	2 (3)	0 (0)	N.A.
Soft tissue	5 (7)	3 (2)	**0.029**
Other	2 (3)	2 (1)	0.273
Osteomedullary only	30 (42)	117 (57)	**0.038**
**Sites of initial metastasis stage 4S**	Stage 4S 26 (100)	Stage 4 ≥ 18 m and MNA 205 (100)	
Bone marrow (cytology)	17 (14)	189 (92)	**<0.001**
Osteomedullary (mIBG scintigraphy)	17 (62)	196 (96)	**<0.001**
Lymph nodes	0 (0)	55 (27)	**<0.001**
Liver	22 (19)	24 (12)	0.101
Brain/spinal cord	0 (0)	9 (4)	N.A.
Lung/pleura	1 (1)	16 (8)	0.008
Skin	2 (3)	0 (0)	N.A.
Soft tissue	0 (0)	3 (2)	N.A.
Other	0 (0)	2 (1)	N.A.
Osteomedullary only	3 (10)	117 (57)	**<0.001**
**Tumor marker (NSE)**	167 (100)	188 (100)	
Normal	4 (2)	1 (1)	0.192
abnormal	163 (98)	187 (99)	
**Tumor marker (VMA/HVA)**	190 (100)	205 (100)	
normal	59 (31)	39 (19)	**0.007**
Abnormal	131 (69)	166 (81)	
**Tumor marker LDH**	185 (100)	202 (100)	
Normal	8 (4)	1 (0)	**0.016**
abnormal	177 (96)	201 (100)	
**Tumor marker ferritin**	142 (100)	166	
Normal	76 (53)	59 (35)	**0.002**
Abnormal	66 (47)	107 (65)	
**Histology (Shimada)**	163 (100)	164 (100)	
Favorable	42 (26)	7 (4)	**<0.001**
Unfavorable	121 (74)	157 (96)	
**Chromosome 1p aberration**	175 (100)	188 (100)	
Heterozygosity	25 (14)	32 (17)	0.155
Imbalance	25 (14)	39 (21)	
Deletion	125 (71)	117 (62)	
**Mutation RAS/p53**	87 (100)	49 (100)	0.081
yes	21 (24)	19 (39)	
**Treatment protocol**	190 (100)	205 (100)	
NB 97	57 (30)	47 (23)	0.137
NB 2004	133 (70)	158 (77)	
Surgery *	190 (100)	205 (100)	
Complete resection	90 (47)	95 (46)	0.960
Incomplete resection	66 (35)	71 (35)	
Biopsy only	19 (10)	21 (10)	
No surgery	15 (8)	18 (9)	
**Radiotherapy**	190 (100)	205 (100)	
Not given	177 (93)	184 (90)	0.282
Given	13 (7)	21 (10)	
**mIBG therapy**	190 (100)	205 (100)	
Not given	171 (90)	43 (21)	**0.003**
Given	19 (10)	162 (79)	
**ASCT**	190 (100)	205 (100)	
Given	151 (79.5)	172 (84)	0.243
Not given	39 (20.5)	33 (16)	
**Antibody therapy**	190 (100)	205 (100)	
Given	30 (16)	24 (12)	0.245
Not given	160 (84)	181 (88)	
**Treatment modalities**	190 (100)	205 (100)	
Neither surgery nor CT	1 (1)	0 (0)	0.294
Surgery only	1 (1)	0 (0)	
CT only	14 (7)	18 (9)	
Surgery and CT	174 (92)	187 (91)	
**Follow-up (years)**			
Median [inter-quartile range; min–max]	4.1 [1.2–11.4; 0.0–22.5]	2.7 [1.2–6.3; 0.0–22.2]	**0.018**
**Cause of death**	83 (100)	133 (100)	
Tumor	72 (87)	116 (87)	0.333
Toxicity	11 (13)	14 (11)	
Tumor or toxicity	0 (0)	3 (2)	

ASCT: high-dose chemotherapy with autologous blood stem cell transplantation. LDH: lactate dehydrogenase. m: months. mIBG: metaiodinebenzylguanidine (scintigraphy, therapy). *N*: number of patients per group. N.A.: not applicable. NSE: neuron-specific enolase. VMA/HVA: vanillylmandelic acid and/or homovanillic acid in urine. % percent of patients per group. * Surgery performed before first recurrence; best result if >1 operation. ** Mann Whitney test.

**Table 2:** Type of mutations in the study group (*N* = 22 in 21 patients) and the control group (*N* = 22 in 19 patients).

Gene	Study Group	Control Group
ALK mutations	8 *	7 #
ALK amplifications	4	3
HRAS	1	1 #
NRAS	1	0
KRAS	0	1
NF1	1	1#
LIN28B	1	0
CCND1	1	0
FGFR1	0	1
PTPN11	0	1
TP53	3 *	0
ATM	1	0
MDM2	1	2
MDM4	0	2 #
CDKN2A	0	2 #
CREBBP	0	1

* In the study group, one case harbored two mutations (ALK and TP53). # In the control group, three cases harbored two mutations (ALK and MDM4; ALK and CDKN2A; HRAS and NF1).

**Table 3:** Multivariable analysis of time-independent and time-dependent risk factors of the study group patients.

Variable		*N* (No Event)	*N* (Event)	HR	95% CI	*p*
	*Model 1: Accounting for mutation status (‘mutation status available’)*					
	**Event-free survival (EFS)** proportional hazard assumption *p* = 0.274					
Mutation	No mutation	37	29	1		
	**Mutation**	**4**	**17**	**2.904**	**1.577–5.348**	**0.001**
Age	<18 months	54	70	1		
	≥18 months	36	30	2.197	0.927–5.203	0.074
ASCT	No ASCT	10	29	1		
	ASCT	80	71	2.697	0.858–8.482	0.090
	**Overall survival (OS)** proportional hazard assumption *p* = 0.229					
Mutation	No mutation	**45**	**21**	**1**		
	**Mutation**	**4**	**17**	**3.214**	**1.678–6.156**	**<0.001**
Best surgery	Complete	65	25	1		
	**Incomplete/biopsy/none**	**42**	**58**	**2.564**	**1.240–5.304**	**0.011**
	*Model 2: Mutation status not included into multivariable modelling* *(‘mutation status not available’)*					
	**Event-free survival (EFS)** proportional hazard assumption *p* = 0.557					
Stage	Stage 1, 2, 3, 4S	64	55	1		
	**Stage 4**	**26**	**45**	**1.609**	**1.082–2.393**	**0.019**
Best surgery	Complete	56	34	1		
	**Incomplete/biopsy/none**	**34**	**66**	**1.923**	**1.255–2.947**	**0.003**
	**Overall survival (OS)** proportional hazard assumption *p* = 0.837					
Stage	Stage 1, 2, 3, 4S	73	46	1		
	**Stage 4**	**34**	**37**	**1.570**	**1.017–2.425**	**0.042**
Best surgery	Complete	65	25	1		
	**Incomplete/biopsy/none**	**42**	**58**	**2.425**	**1.504–3.909**	**<0.001**

**Table 4:** Recurrence patterns of study and control group patients.

Parameter	Study Group	Control Group	*p*
	Stages 1, 2, 3, 4S, 4 < 18 m	Stage 4 ≥ 18 m and MNA	
	*N* (%)	*N* (%)	
**Recurrences**	88 (100)	139 (100)	
Primary site	58 (66)	84 (60)	**0.036**
Osteomedullary *	31 (35)	80 (58)	**0.001**
Bone marrow	20 (23)	62 (45)	**0.001**
Lymph nodes	13 (15)	11 (8)	0.124
Liver	18 (20)	19 (14)	0.202
Brain/spinal cord	18 (20)	21 (15)	0.368
Lung/pleura	6 (7)	8 (6)	0.783
Skin	0 (0)	0 (0)	N.A.
Soft tissue	0 (0)	2 (1)	N.A.
Other	4 (5)	4 (3)	0.714
Osteomedullary only	6 (7)	48 (35)	**<0.001**
Primary site only	32 (36)	29 (21)	**0.014**
**Number of recurrence sites**			
1	49 (55)	59 (42)	0.077
>1	40 (45)	80 (58)	
Median time ** (d) **min–max**	382 28–3407	455 60–3427	**0.003**
**EFS and OS**			
5y-secEFS *** (%)	13.0 (5.6–20.4)	10.2 (5.0–15.4)	**0.005 ^†^**
5y-secOS (%)	14.4 (6.6–22.2)	9.8 (4.6–15.0)	0.124 ^†^
10y-secEFS (%)	13.0 (5.6–20.4)	10.2 (5.0–15.4)	**0.005 ^†^**
10y-secOS (%)	14.4 (6.6–22.2)	9.8 (4.6–15.0)	0.124 ^†^

^†^ Generalized Wilcoxon test; * by mIBG scintigraphy; ** time from first diagnosis to first recurrence (days); *** secondary EFS; Secondary malignancy: 0 (exclusion criterium); Ganglioneuroma: 0.

## Data Availability

All clinical data are contained within the article or the supplementary material. Molecular data are available from the authors upon reasonable request.

## Supplementary Materials

The following are available online at https://www.mdpi.com/article/10.3390/cancers13174360/s1, Figure S1: EFS and OS of 190 study group patients with *MYCN* amplification by stage (1, 2, 3, 4S vs. stage 4 aged <18 months), Figure S2: EFS and OS of 185 study group patients by stage and best result of surgical resection, Figure S3: EFS and OS of 87 study group patients by independent risk factors ‘presence of mutation’ and ‘best result of surgical resection’ *.
