# Supplementary material for: Genetic Alterations and Resectability Predict Outcome in Patients with Neuroblastoma Assigned to High-Risk Solely by MYCN Amplification
Source: Cancers (Basel). 2021 Aug 28;13(17):4360. doi: 10.3390/cancers13174360 (PMC8430929; doi:10.3390/cancers13174360)
Supplement: Supplementary file 1 [file cancers-13-04360-s001.zip › cancers-1332955-supplementary.pdf]

Supplementary Material

Supplementary Figure S1

EFS and OS of 190 study group patients with *MYCN* amplification by stage (1, 2, 3, 4S vs. stage 4 aged <18 months)

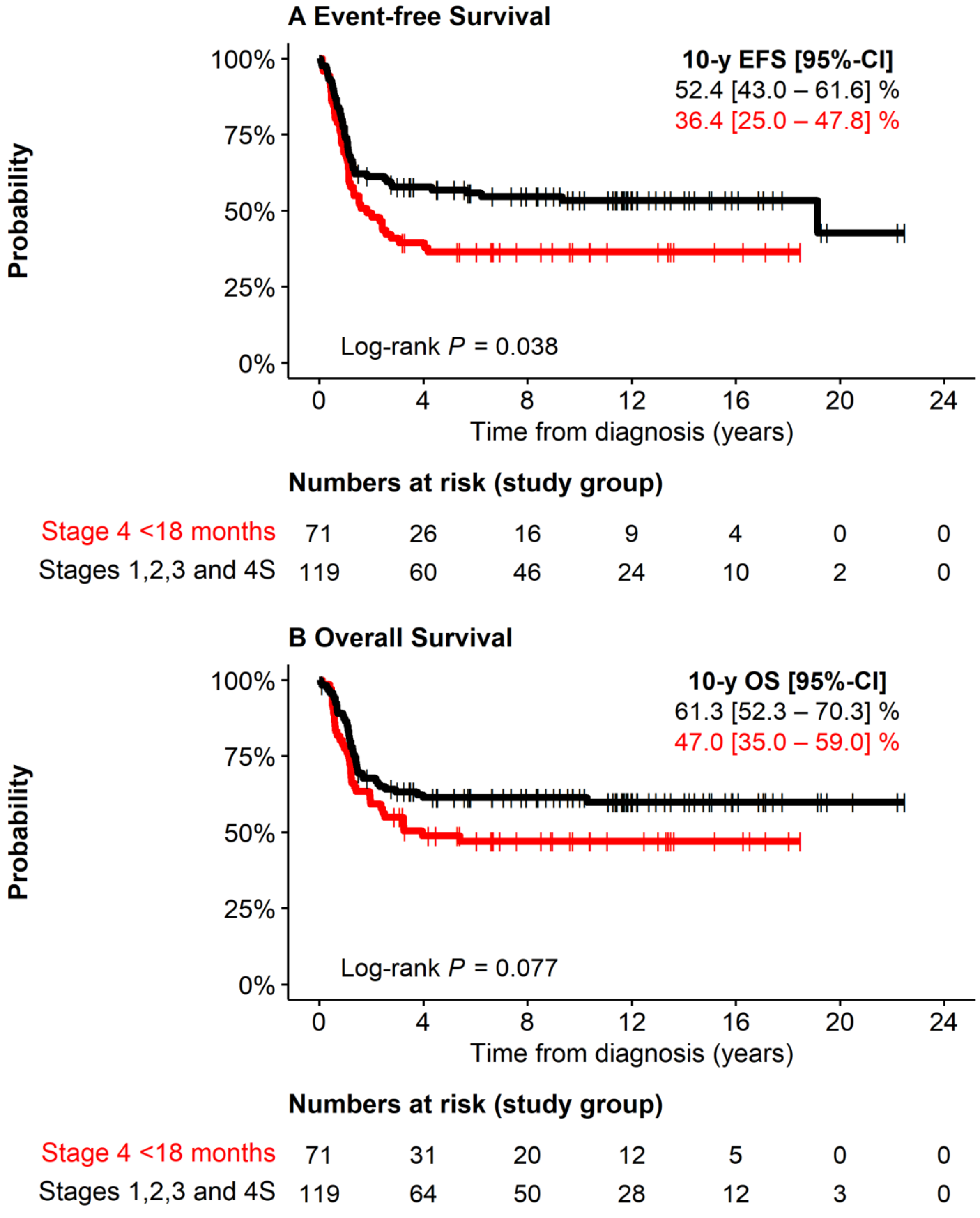

Supplementary Figure S2
EFS and OS of 185 study group patients by stage and best result of surgical resection

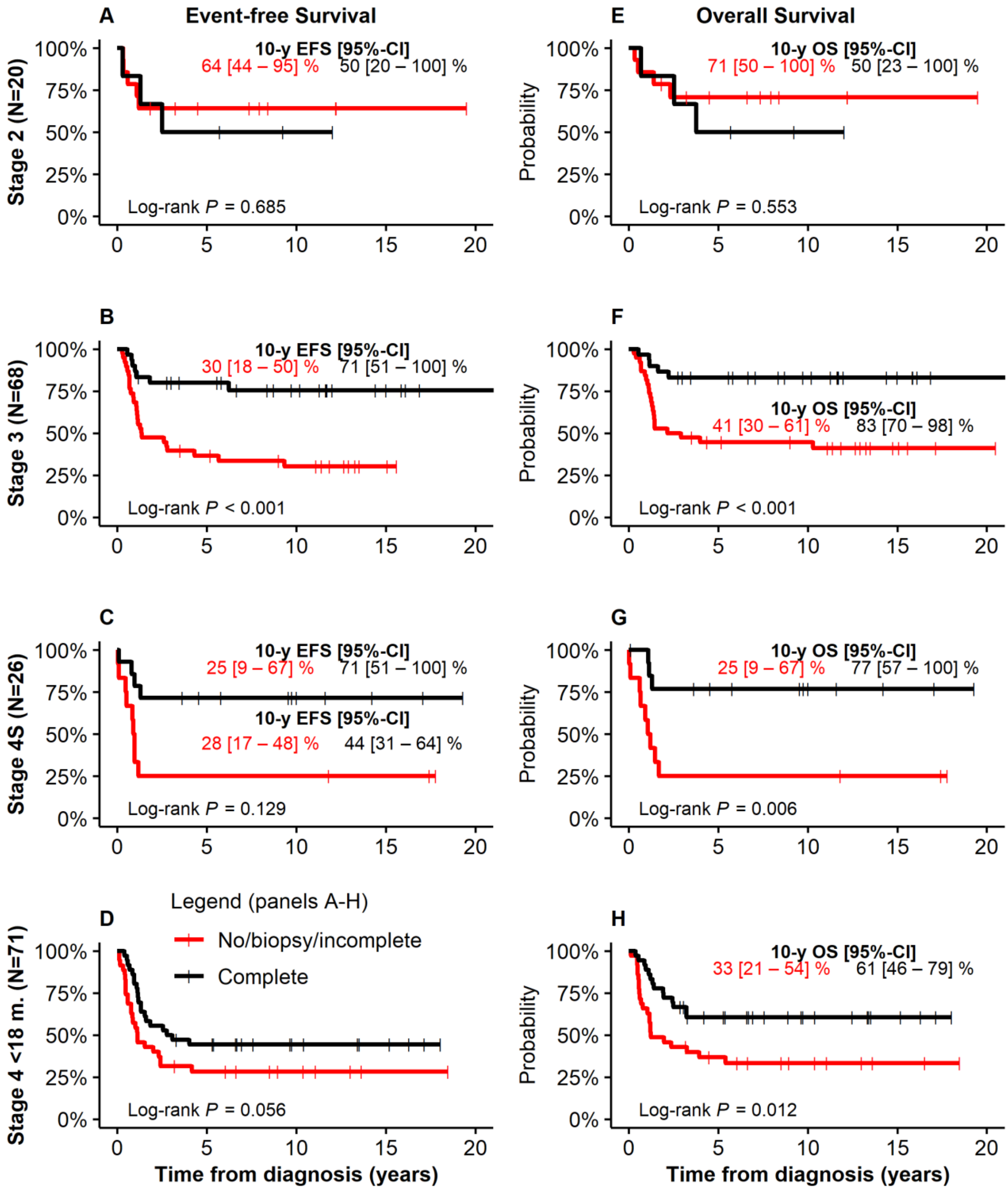

Supplementary Figure S3

EFS and OS of 87 study group patients by independent risk factors ‘presence of mutation’ (mutation vs. no mutation) and ‘best result of surgical resection’* (no/incomplete/biopsy vs. complete)

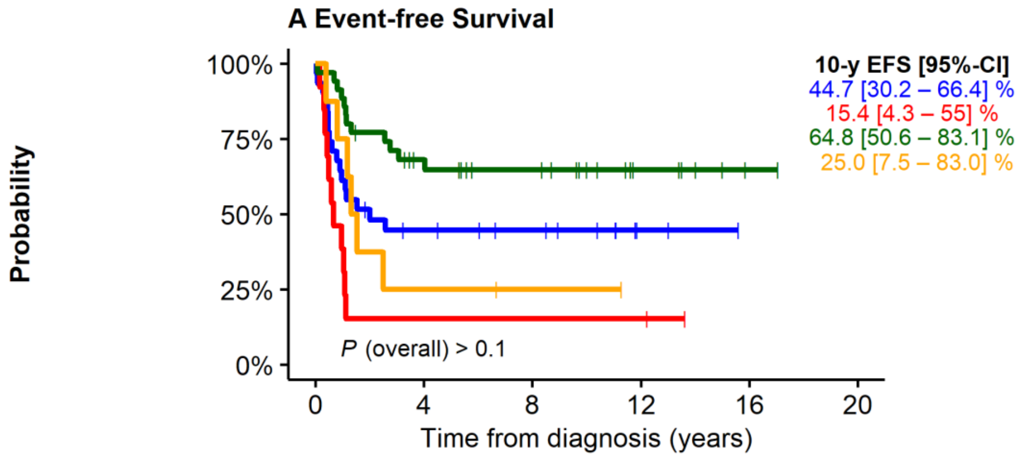

Numbers at risk (study group)

	0	4	8	12	16	20
No/incomplete/ biopsy, no mut.	31	12	9	2	0	0
No/incomplete/ biopsy, mut.	13	2	2	2	0	0
Complete, no mut.	35	20	15	6	1	0
Complete, mutation	8	2	1	0	0	0

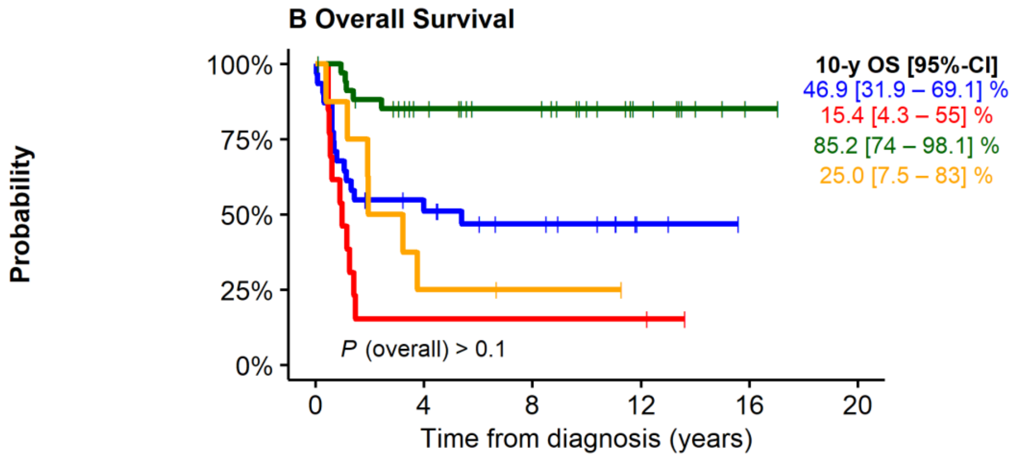

Numbers at risk (study group)

	0	4	8	12	16	20
No/incomplete/ biopsy, no mut.	31	14	9	2	0	0
No/incomplete/ biopsy, mut.	13	2	2	2	0	0
Complete, no mut.	35	23	18	8	1	0
Complete, mutation	8	2	1	0	0	0

*If more than one surgical intervention, the best result was counted.

Pairwise comparisons (Log-rank p-values, Bonferroni-Holm adjusted)

EFS:

No/incomplete/biopsy, no mutation vs. Complete, no mutation: 0.059

No/incomplete/biopsy, no mutation vs. No/incomplete/biopsy, mutation: 0.059

No/incomplete/biopsy, no mutation vs. Complete, mutation: N.A. (curves cross)
Complete, no mutation vs. No/incomplete/biopsy, mutation: < 0.001
Complete, no mutation vs. Complete, mutation: 0.057
No/incomplete/biopsy, mutation vs. Complete, mutation: 0.155

OS:
No/incomplete/biopsy, no mutation vs. Complete, no mutation: 0.002
No/incomplete/biopsy, no mutation vs. No/incomplete/biopsy, mutation: 0.065
No/incomplete/biopsy, no mutation vs. Complete, mutation: N.A. (curves cross)
Complete, no mutation vs. No/incomplete/biopsy, mutation: < 0.001
Complete, no mutation vs. Complete, mutation: 0.001
No/incomplete/biopsy, mutation vs. Complete, mutation: 0.164
